# Preceptors’ and nursing students’ experiences of collaborative pedagogical strategies in home healthcare: a qualitative study

**DOI:** 10.1186/s12912-026-05651-3

**Published:** 2026-11-11

**Authors:** Mariette Bengtsson, Elin Taube, Elisabeth Carlson

**Affiliations:** Department of Care Science, Faculty of health and society, Malmö University, Malmö, SE205 06 Sweden

**Keywords:** Clinical learning, Clinical training, Collaborative learning, Home healthcare, Nursing students, Preceptors, Structured learning activities

## Abstract

**Background:**

In recent years, healthcare organizations have globally undergone a transition from hospital-based care to primary and municipal care settings. This transition has created new expectations for how future nurses deliver care in non-hospital settings, including patients’ homes. Therefore, pedagogical strategies that effectively support nursing students’ clinical training in home healthcare environments are essential. This study aimed to explore preceptors’ and nursing students’ experiences of using collaborative pedagogical strategies during clinical training in home healthcare.

**Methods:**

Data were generated through paired discussions with 60 nursing students and their preceptors during clinical training in municipal home healthcare in Sweden between 2022 and 2024. In addition, four focus-group discussions, two with students and two with preceptors, were conducted separately to reduce social pressure and deepen the data. All discussions were recorded, transcribed, and analysed as a single unit using thematic analysis.

**Results:**

Preceptors and nursing students perceived that using collaborative strategies in home healthcare enhanced learning by promoting reflective dialogue, supporting professional development, and fostering students’ independence over time. These findings illustrate how collaboration between students and preceptors strengthens clinical training in home healthcare based on three interrelated organizational themes: • Team‑based interaction for learning and support refers to a collaborative learning unit in which students and preceptors engage in shared activities that foster a supportive, reflective, and competence‑enhancing learning environment. • Tasks for bridging theory and practice refers to the importance of structured activities and guided reflection in collaborative clinical learning units for bridging theoretical knowledge with clinical application. • Training and precepting in relation to the length of the collaboration refers to how the clinical training duration and environment influence the teamwork, depth of learning, autonomy, and pedagogical strategies.

**Conclusion:**

Home healthcare offers a unique and important setting for nursing students’ clinical training. From a pedagogical standpoint, well-organized placements that incorporate collaborative strategies with structured learning activities could prepare students for the growing patient population in home healthcare and may positively influence career choices. Core collaborative pedagogical components include team-based learning and reflective practices embedded in everyday nursing care.

**Clinical trial number:**

Not applicable

**Supplementary Information:**

The online version contains supplementary material available at https://doi.org/10.1186/s12912-026-05651-3.

## Background

Over the past decade, healthcare has globally shifted from hospital-based to home-based care [1, 2]. In Europe, this reorientation has enabled municipal registered nurses to provide more complex healthcare interventions outside traditional hospital settings [3, 4, 5], including both basic nursing procedures (e.g. wound care, vital signs monitoring, and blood sampling) and more advanced treatments (e.g. medication management and dialysis). As a result, patients can remain within their familiar and supportive environments while still receiving high‑quality clinically appropriate medical treatment and nursing care [4].

Patients who receive home healthcare represent a highly heterogeneous group in terms of age, living condition, diagnosis, and nursing needs. This diversity contributes to a level of complexity which may differ from other settings. Registered nurses working with municipal home healthcare are required to work more closely with next of kin alongside the patients themselves, thereby encountering additional ethical dimensions concerning restrictions, confidentiality, and the negotiation of patients’ preferences [6, 7]. In addition, the autonomous nature of home healthcare requires registered nurses to practice a high degree of independent judgment and clinical decision-making in real time [8]. Their assignments are further complicated by time pressures, unpredictable working conditions, and the need to coordinate healthcare activities across multiple organizations [9]. Due to their particularly demanding role, municipal registered nurses need to possess advanced clinical competencies that encompass theoretical and practical knowledge, critical reasoning, and the ability to perform complex interventions.

To ensure safe and high‑quality care in patients’ homes, registered nurses must support one another in handling demanding tasks and collaborate closely with a range of healthcare professionals [10]. Accordingly, they need strong collaborative skills to coordinate care across professional boundaries [10, 11]. Although such skills are cultivated during their nursing education training on campus [12, 13], the integration of theory into practice is most effectively achieved through direct patient encounters during clinical training, where students must navigate real-time challenges, collaborate with healthcare teams, and respond to individual patient needs [14, 15]. Clinical training in diverse clinical contexts provides nursing students with authentic experiences that foster professional identity, critical thinking, and adaptability [16]. To equip graduates with the competencies necessary to deliver safe, high-quality care, nursing education must provide clinical training across a broader range of clinical settings, including patients’ homes [9].

Despite its learning potential, the patient’s home can be a challenging pedagogical learning environment due to the complexity of home healthcare and the contextual demands of the home setting [17]. To address the pedagogical challenges, preceptors could explore collaborative learning models used in other healthcare settings and assess their applicability to the home healthcare context [18]. *Collaborative learning* is an umbrella term that includes both formal and informal pedagogical strategies through which two or more students learn alongside each other while being supervised by a preceptor. They may involve two students working with one preceptor (the two-to-one precepting model) or, more broadly, small groups of students interacting with each other. These strategies enable nursing students to optimize their own and each other’s theoretical knowledge and clinical skills [19, 20]. Collaborative learning is grounded in sociocultural learning [21], in which students develop knowledge both from and with each other through joint activities and shared reasoning [19, 20]. This can be further understood through Vygotsky’s theory regarding the Zone of Proximal Development, which describes the distance between what students can achieve independently and what they can accomplish with collaboration and peer support. Based on this theory, scaffolding and joint problem‑solving in collaborative learning situations provide opportunities for students to extend their capabilities. The core pedagogical elements in collaborative learning include cooperation, communication, and reflective practice. Collaborative learning also incorporates structured learning activities, which enable students to work together on different tasks without immediate intervention from the preceptor. Instead, the preceptor takes on a more observational and reflective role [22, 23]. When peer collaboration is not feasible, students can conduct these structured learning activities individually.

Research indicates that collaborative learning strengthens nursing students’ training in different clinical settings and supports their professional growth [24]. However, its suitability for clinical placement in patients’ homes is a relatively underexplored area. Studies on a dedicated educational unit in municipal home healthcare [25] and on related care contexts, such as primary care [26] and municipal elderly care [27], offer some insights. To ensure competence and meet the demands placed on future nurses, research should identify new strategies and preceptorship models tailored specifically to the conditions of home healthcare. Such research should explore both students’ and preceptors’ perspectives on learning and precepting, as these insights are crucial for designing pedagogical models that are feasible, evidence‑based, and responsive to the realities of care in patients’ homes. Research should also identify strategies that enable students to translate theoretical knowledge into clinical practice and to support students’ development into professional nurses.

## Methods

This study aimed to explore preceptors’ and nursing students’ experiences of using collaborative pedagogical strategies during clinical training in home healthcare.

Adopting a pragmatic perspective, this study defines *collaborative pedagogical strategies* as all activities, structures, and interactions that support nursing students’ learning and professional development through shared engagement [18]. Grounded in sociocultural learning theory, these strategies aim to promote the integration of the practical, cognitive, and affective domains of learning [21].

In this study, *clinical training* refers to patient-care activities that allow nursing students to apply theoretical knowledge and further develop their cognitive skills in problem solving, critical thinking, clinical decision making, clinical reasoning, and clinical judgment within authentic clinical settings [28]. The study focuses on home healthcare as the *clinical learning environment*, which is the psychological, social, cultural, and physical context in which learning occurs, shaping how students perceive, engage with, and make meaning of their clinical experiences [29].

### Design

This qualitative study adopted a pedagogical perspective. Data were collected through paired discussions [30] involving students and their preceptors and through focus-group discussions [31] with students and preceptors separately. Data collection took place in different municipal home healthcare settings affiliated with a university in southern Sweden over the course of four semesters, from spring 2022 to spring 2024.

### Setting

The Swedish nursing programme is a three-year bachelor’s programme comprising 180 European Credit Transfer and Accumulation System credits. This study focused on nursing students’ clinical training in municipal home healthcare during the students’ sixth and last semester in the programme at a university in Southern Sweden. In the autumn of 2021, the nature of the students’ home healthcare placement was revised, and its duration was extended from eight days to four weeks. To support the transition, the university offered the preceptors a day-and-a-half introductory course coordinated by the first author (MB) and partly facilitated by the last (EC). The course focused on general pedagogical strategies for preceptorship, including the use of collaborative learning models [32], reflection methods [33, 34], and formative assessment [35]. To provide preceptors with tools to facilitate student learning in a structured, research-based, and pedagogical manner, the course provided an overview of tasks for students to explore individually or with a peer during clinical training. This overview was developed by experienced university faculty members and addressed typical scenarios encountered in home healthcare. In particular, it introduced a list of 15 structured learning activities with clear guidelines. These activities all comprised four key components: planning, execution, reflection, and follow‑up. The nursing process itself constituted one of these structured learning activities and was the only mandatory activity whose application and outcomes were part of the students’ clinical assessment. Supplementary Files 1 and 2 provide an overview of the structured learning activities and an example illustrating their application using the nursing process.

Prior to entering the clinical training, all nursing students participated in a mandatory three-hour introductory session about care in patients’ homes. The session focused on patient-centred care, patient safety, interprofessional collaboration, and relevant clinical nursing skills. The students were also introduced to the aforementioned overview of tasks, including the mandatory nursing process.

### Participants

Nursing students and preceptors were recruited from municipal home healthcare placements in southern Sweden. The recruitment for the paired discussions sought to include a diversity of perspectives in a pre-determined sample size. The sample size was guided by the concept of ‘information power’, considering the study aim, sample specificity, theoretical framework, quality of dialogue, and analytical strategy [36, 37]. Accordingly, a sample of 60 nursing students (around 15 students per semester) and their preceptors was considered adequate. Following the confirmation of clinical placement schedules, students from four programme semesters (approximately 400 students in total) and their preceptors were randomly selected and invited to participate via email. A consecutive sampling strategy was then applied whereby the first 15 respondents from each programme semester, together with their respective preceptors, were included in the study. The inclusion criteria for the preceptors were (1) the completion of the above-described introduction course on student supervision; (2) a minimum of six months’ experience as a registered nurse in home healthcare; and (3) prior experience in precepting nursing students. Of the participating preceptors, 53 identified as women and 7 as men. Among the students, 56 were women and 4 were men. Consistent with the study aim, no additional demographic data were collected; the focus was on variations in precepting practices rather than on differences in professional experience or organizational context.

The focus-group participants were recruited among those who had taken part in the paired discussions. The focus-group sample consisted of five students and nine preceptors. The students were divided into two focus groups of three and two participants, and the preceptors into two focus groups of four and five participants.

### Data collection

All data were collected through paired or focus-group discussions after the students had completed their clinical placement and final assessment. Pair and group discussions were chosen over individual interviews to facilitate participant interaction, stimulate reflection, enrich accounts through shared elaboration, and address memory gaps, thereby generating rich and nuanced data [30, 31, 38]. Since potential hierarchical influences and social desirability could shape participants’ responses in the paired discussions, homogeneous focus groups—comprising only students or only preceptors—were conducted to promote honest and spontaneous reflections [31]. The conversational format enabled the participants to reflect on shared experiences of learning and precepting and to co-construct understandings of the precepting process in home healthcare. The participants were actively encouraged to speak freely, and the interviewers ensured that no single participant dominated the discussions. Each discussion concluded with a summary based on the field notes, enabling participants to confirm or clarify the discussed content.

The first author (MB) facilitated the pair discussions and took field notes. These discussions lasted approximately 30 min. The focus-group discussions were facilitated by the first and second authors and guided by a semi-structured interview guide (Supplementary File 3) [38]. The guide was developed based on themes emerging from the paired discussions and included open-ended and follow-up questions related to the following themes: precepting process, collaboration, reflection, learning environment, structured learning activities, and overall placement experience. The second author (ET) moderated the focus-group discussions and used probing questions, such as ‘Can you elaborate on that?’ and ‘Could you clarify what you mean?’, to deepen the participants’ reflections, while the first author (MB) took field notes. The focus groups were conducted at the university and lasted 20 to 105 min (mean 45 min). All discussions were recorded and transcribed by the first and second authors (MB and ET), allowing for close engagement with the data and ensuring a transparent and traceable analytical process grounded in the participants’ own narratives.

### Analysis

The data collected from the paired and focus-group discussions were analysed as a single dataset, while each discussion was also reviewed individually to capture variation in experiences and perspectives. The thematic analysis was guided by Braun and Clarke’s six-phase approach [39], with themes developed at the semantic level. The analytical process commenced during data collection through the first author’s (MB) field notes; information power was continuously evaluated during data collection by assessing the extent to which successive discussions within the research team contributed novel insights relevant to the study aim. Although the participants reported diverse experiences, substantial convergence was evident in their descriptions of preceptorship in home healthcare. The richness, recurrence, and relevance of the data indicated sufficient information power to address the study aim [36, 37]. In addition, no substantially new insights were identified, developed, or constructed during the final phase of data collection.

To familiarize with the data, the first (MB) and second (ET) author repeatedly read the transcripts, focusing on what the students and preceptors revealed in relation to the aim (Phase 1). During this process, ideas for coding and reflections were noted, and the data were manually coded using coloured highlights to identify patterns and collate relevant extracts within categories that were further developed in subsequent stages (Phase 2). Data were systematically grouped into themes that addressed the study aim. This phase involved organizing and iteratively sorting codes into thematic clusters using separate sheets, allowing for the comparison, refinement, and development of coherent overarching themes (Phase 3). Examples of the coding are shown in Table 1. Thereafter, the themes that were identified in Phase 3 were reviewed by gathering, re-evaluating, and modifying all data relevant to each theme. This phase ended with the development of a clear understanding of the themes, their relationships, and the overall narrative conveyed by the data (Phase 4). Finally, the themes were defined to capture their essence. Each theme represented a distinct aspect of the data, with extracts organized into coherent and internally consistent accounts (Phase 5). This included checking that the themes worked in relation to the coded extracts (Level 1) and the entire dataset (Level 2), resulting in a thematic map of the analysis. The results are presented to convey the complexity of the data in a coherent and meaningful way (Phase 6). Throughout these final phases, all authors discussed the themes and their interpretations and reached consensus regarding the final themes and results. There were no disagreements among the authors, as the participants expressed largely consistent views with limited variation in their accounts. Quotations are included in the results section to provide transparency regarding data interpretation and to exemplify the findings, emphasizing shared experiences and recurring patterns across participants. Care has been taken to ensure that the selected quotations are representative of the broader dataset.

**Table 1:** Example of data extract, with codes applied

Data extract	Coded for
*Student 1*: From my perspective, the team-based preceptorship model worked well compared to the traditional one-to-one model. We worked in different constellations, which supported my learning	Team-based preceptorship model - learning (student)
*Preceptor 3*: Honestly, I found that the team setup made my role more flexible. Instead of always being tied to two students we could adjust our precepting depending on the situation and the students’ needs, which felt more realistic in the context of home healthcare.	Team-based preceptorship model - support (preceptor)
*Student 39*: I worked with a structured learning activity on wound care, on my own when my preceptor was busy. I learned a lot about different types of dressings, and even though I used to find wounds unpleasant, I now feel much more comfortable handling them.	Task - Structured learning activity – working alone, useful (student)
*Preceptor 46*: Over time, I’ve seen my students grow more confident and independent. Toward the end of the placement, they were able to carry out own visits to patients. I trusted them. They had developed in both skills and professional judgement.	Develop over time - independent (Student)

### Rigour

To ensure trustworthiness, the study adhered to the criteria of credibility, transferability, dependability, and confirmability outlined by Guba and Lincoln [40]. Braun and Clarke’s thematic analysis was chosen for its systematic yet flexible approach to identifying, analysing, and reporting patterns within qualitative data [39]. The analysis was conducted inductively, allowing themes to be identified form the data rather than being predetermined, thereby enhancing the credibility and authenticity of the findings. Moreover, the study’s credibility and trustworthiness were strengthened through iterative discussions among all authors, who jointly reviewed, challenged, and refined the themes until consensus was reached. This collaborative process also enhanced dependability and confirmability, as analytical decisions were critically examined and agreed upon by all authors.

Recognizing that the quality of qualitative research is influenced by their’ competence and reflexivity, the authors employed several strategies to enhance trustworthiness. The research team comprised researchers with expertise in nursing, education, pedagogy, and qualitative methodology who contributed to different phases of the study to varying degrees. This combination of disciplinary and methodological expertise contributed to a nuanced understanding of both the study context and the collected data; it also facilitated critical reflection and the exploration of alternative interpretations [37]. For instance, the paired discussions were conducted by the first author (MB), who had experience in collaborative learning in home healthcare; in contrast, the focus-group discussions were moderated by the second author (ET), who had no prior experience with collaborative learning or home healthcare, thereby contributing an alternative perspective and reducing the risk of assumptions shaping data collection. Furthermore, the last author (EC), who has expertise in pedagogy and was not involved in the data collection, contributed to the analysis and provided an additional analytical perspective. Although the first author (MB) had previously assessed some of the student participants and two authors (MB, EC) had participated in the preceptors’ introductory course, there was no evidence that these prior relationships influenced data collection. The second author (ET) had no prior relationship with the participants. Nevertheless, the authors acknowledge that their professional backgrounds and pre-understandings may have influenced the analytical process. To address this issue, the authors maintained reflexivity throughout the study by continuously engaging in critical dialogue, questioning their assumptions, and considering alternative interpretations.

To enhance transferability, the study provides rich descriptions of the study context and data collection procedures, enabling readers to assess the applicability of the findings to other contexts. Nevertheless, the absence of the participants’ characteristics may limit the study’s transferability. Another potential limitation concerns the data collection methods: Individual interviews might have generated more critical or sensitive accounts and prevented the potential socially desirable responses in the paired discussions. However, paired and focus-group discussions were considered more appropriate for the study aim, as they promoted interaction and collective reflection on shared experiences. The use of complementary data collection methods also enabled triangulation and enhanced credibility; the focus-group discussions confirmed and elaborated on the patterns identified in the paired discussions. A further constraint was student participation, which was limited because the data collection coincided with the final stage of the nursing programme, when students were completing examinations and degree requirements. Moreover, as participation was voluntary, there is a risk that the study included students specifically interested in their education, thus excluding more critical voices. While the participants were recruited from several municipalities and clinical placements, they were all affiliated with the same university and geographical region. Nevertheless, the findings demonstrated considerable variation in the application of collaborative learning activities in home healthcare settings, supporting not only the relevance of the findings to the study aim but also transferability.

### Ethical considerations

The study did not require a clinical trial registration. Ethical approval was obtained from the Swedish Ethical Review Authority (Dnr 2020–06858), and permission to carry out the research was granted by the head of the nursing department at the university as well as by the operational managers at the clinical placement sites. Furthermore, the study was conducted in accordance with the Declaration of Helsinki [41]. All participants received both oral and written information about the study and gave their consent, and the study posed no risk of harm to the participants. Consent for publication was not applicable, as the manuscript does not contain any identifiable participant information. To protect the participants’ confidentiality, the authors ensured that the findings cannot be linked to any specific workplace, individual student, or preceptor. Therefore, only minimal demographic data were collected, and the quotes included in the results section were anonymized.

## Results

Examining the perspectives of both preceptors and nursing students, the thematic analysis identified patterns that illuminate collaborative clinical training in home healthcare. Overall, the training included a range of precepting models in which all students had a designated preceptor responsible for follow-up and assessment. The preceptors and students indicated that collaborative strategies within the home healthcare context facilitated learning for both parties by promoting professional development and fostering student independence. However, the preceptors encountered some challenges when it came to balancing their precepting role with the dynamic of being a guest in patients’ homes, acknowledging the patients’ living conditions and illness and maintaining their safety. Organizational conditions, together with the interpersonal interactions among preceptors and students, shaped how collaborative clinical training is enacted in home healthcare. Using collaborative strategies, both students and preceptors navigated a dynamic learning process and strengthened the conditions necessary to achieve the intended learning outcomes.

The analysis resulted in three themes. *Team‑based interaction for learning and support* refers to a collaborative learning unit in which students and preceptors engage in shared activities that could foster a supportive and competence‑enhancing learning environment for both preceptors and students. *Tasks for bridging theory and practice* refers to the importance of structured activities and reflection in collaborative learning units for bridging theoretical knowledge with clinical application. For students, these activities supported critical thinking and the practical application of theoretical concepts, while for preceptors, they promoted reflective teaching and created opportunities to further develop their pedagogical and clinical competence. *Training and precepting in relation to the length of the collaboration* refers to how the duration and structure of the training environment influenced teamwork and pedagogical strategies. In particular, they supported students’ depth of learning, autonomy, and progressive development of clinical competence while also supporting a more adaptive and reflective precepting approach among preceptors. Together, these themes indicate the value of structured collaborative strategies in enhancing both pedagogical effectiveness and professional development within the context of home healthcare. The themes are further elaborated below to capture the nuanced experiences and strategies used during clinical training.

### Team‑based interaction for learning and support

Overall, most preceptors organized their preceptorship, either informally or formally, into collaborative units, and preceptors and students variably used collaborative strategies during the clinical training. They both recognized the organizational benefits of a team-based preceptorship model compared to the traditional one-to-one preceptorship model. However, they agreed that team alternatives were needed because the traditional peer-learning team structure (two-to-one model) did not always align with the work in home healthcare, the preceptors’ working conditions, or the learning needs of individual students. For instance, having multiple individuals present in patients’ homes posed challenges, and family members expressed their own concerns. Therefore, the preceptors created alternative team constellations that differed from traditional peer‑learning teams and better suited students’ learning and preceptors’ care responsibility in home healthcare. These constellations varied and included the following teams: (a) one preceptor responsible for two collaborating students, (b) two preceptors alternately responsible for two collaborating students, (c) two students collaborating with two non-collaborating preceptors, (d) three students collaborating with non- or somewhat-collaborating preceptors, and (e) one student collaborating with two or more preceptors due to lack of peers to collaborate with. Most preceptors favoured this team-based approach:

When we adjusted the team constellations, it became easier for me to balance my responsibilities, keep patients safe, and provide more flexible learning opportunities for students that fit the realities of the workplace (Preceptor 24)

The students agreed with this approach:

Working in more adapted team constellations made it easier for preceptors to ensure patient safety while also supporting our learning (Student 7)

On the other hand, the preceptors also described the complexities involved in precepting students within teams, particularly when differences in knowledge levels and personal characteristics were present. They emphasized that these challenges can be further intensified in patients’ homes, shaped by the limited space and resources and the more constrained scope for training activities.

Working with students in home healthcare requires flexibility. Students’ knowledge levels and personalities may differ considerably, and in a patient’s home, the environment often limits what can be done and how much time can be devoted to supervision. (Preceptor 52)

This highlights how team-based supervision requires ongoing adaptation, as preceptors need to balance individual learning needs while maintaining a cohesive learning environment. Some preceptors reported that they sometimes allowed the students, in pairs or by themselves, to visit patients alone, while still acknowledging their overall responsibility for the safety of the patients. In these cases, the preceptors had obtained the patients’ and their next of kin’s consent in advance and described a deliberate strategy when selecting patients aimed at creating a familiar and secure learning context for the students and the patients:

We mainly selected patients who were well known to both us and the students (Preceptor 8)

This reflects how familiarity was used pedagogically to support students’ confidence and readiness for patient encounters. In some cases, the preceptors even allowed the students to treat new patients if they only required a simple procedure, such as checking blood pressure or taking a blood sample, or if the preceptors were convinced that the students would be able to handle the situation. The students who visited patients without their preceptors reported that they felt valued by the patients and felt part of the clinical teams.

Most of the students mentioned that they appreciated both the benefits of teamwork and the opportunity to work alone with the preceptor.

Working in a team allowed us to learn from each other’s perspectives and support one another, while the one-on-one time with the preceptor made it possible to focus more deeply on my own learning needs and receive more tailored guidance. (Student 2)

This illustrates how combining peer interaction with individualized precepting created complementary learning conditions, supporting both shared understanding and personal development.

In addition, the students also collaborated with other healthcare professionals, including home care staff, mobile care teams, and healthcare professionals from primary care and rehabilitation units:

The clinical environment felt like a learning community, where everyone we interacted with among the staff contributed to our learning”(Student 58)

This collaboration developed through the students’ participation in team rounds and care planning, during both direct patient care and study visits. This strategy was recognized by both preceptors and students as an essential contribution to the students’ learning process and their understanding of the patients’ care needs:

Person-centred care was experienced as dependent on healthcare professionals working closely together, sharing responsibilities, planning jointly, and making decisions collaboratively as a team (Student 44)

Additionally, the interaction between students and other healthcare professionals fostered positive interprofessional relationships. Overall, the students in this study perceived the team environment as collegial and inclusive, which helped foster a sense of belonging and supported their engagement in collaborative learning.

The team-based approach supported preceptors as well as students. Although each student had a designated primary preceptor, preceptorship was mostly allocated to a team rather than an individual preceptor. The preceptors listed several advantages to team-based preceptorship, including the shared responsibility, the reduced burden on individual preceptors, and the increased flexibility, especially during staff shortages. In particular, they valued the continuous support from colleagues, relieving them from feeling alone in undertaking individual responsibility for students. Some preceptors also emphasized the relational dimension of their collaboration, highlighting the value of giving and receiving constructive feedback within the team:

As a preceptor, I see an important part of our role as being critical friends to one another, offering honest feedback and standing up for each other when needed (Preceptor 11)

Thus, collegial support extended beyond practical cooperation, evolving into a form of reflective practice that facilitated feedback, mutual learning, and ongoing professional development.

### Tasks for bridging theory and practice

The students reported that they performed structured learning activities to varying extents:

When the preceptors were busy with activities, we couldn’t take part in, we engaged in structured learning activities, sometimes on our own and sometimes in pairs, which still felt meaningful for our learning (Student 27)

Similarly, the preceptors highlighted how these activities not only supported student learning but also facilitated workflow during demanding periods:

As a preceptor, I found that these structured activities offered valuable learning opportunities for the students and also helped ease the pressure on us during particularly busy periods (Preceptor 36)

This also illustrates that some preceptors and students valued structured learning activities as a tool for integrating knowledge into clinical practice. Nevertheless, not all students shared this view, explaining that performing structured learning activities as additional tasks added stress to their daily routines:

It felt like these structured learning activities just added more to an already busy day without really helping me in the clinical work (Student 60)

This indicates that some students struggled to see the connection between theory and practice; they felt that the activities did not contribute meaningfully to their clinical development and learning and therefore questioned their relevance. The students also pointed out that the collaborative aspects of these activities were dependent on each student’s willingness to engage, listen, and learn from their peers.

As previously mentioned, the structured learning activities included a mandatory activity focusing on the nursing process. Most students found this activity particularly effective in deepening their understanding of the clinical context and patient care. As this activity was a formally assessed component of the education, the preceptors mentioned that they supported students in identifying suitable patients for it, ensuring relevance and applicability.

Moreover, some students and preceptors reported that they had had the opportunity to take part in broader team-based reflective discussions based on the themes outlined in the overview of optional tasks. These discussions involved all members of the student-preceptor group. According to the students, the reflective discussions created a sense of openness in collaborative learning, which allowed them to agree with, challenge, and build upon each other’s ideas:

When we discussed cases together as a whole group, I could hear different perspectives and not just my own preceptor’s views. It really helped me understand the bigger picture and made the learning much richer” (Student 31)

The reflective sessions were beneficial not only for students but also for preceptors, who described these discussions as supportive and enriching:

These group discussions were also valuable for us as preceptors. We learned from each other’s experiences and from how the students approached the situations, which made the discussions rewarding (Preceptor 53)

Thus, the discussions contributed to a shared learning atmosphere within the clinical environment.

In addition, both students and preceptors described how reflections were routinely used before and after patient visits to ensure patient safety and promote learning. Prior to patient visits, the students planned care independently or with peers, and they presented and discussed their plans with their preceptors. The students reported that they felt safe to express ideas and ask questions and highlighted that these discussions enhanced their confidence in managing patient care independently. The preceptors mentioned that they used reflective questioning techniques to stimulate critical thinking and deepen students’ clinical reasoning while simultaneously supporting formative feedback:

I tried to ask questions instead of giving answers, encouraging them to think things through themselves and feel free to explore their own, sometimes creative, ways of caring (Preceptor 22)

The use of such techniques reflected how some preceptors fostered an open learning climate in which students were encouraged to think independently and explore alternative approaches to patient care.

### Training and precepting in relation to the length of the collaboration

According to both preceptors and students, the initial phase of the clinical training period, typically the first two weeks, was characterized by learning through observation and imitation. During this period, the students closely followed their preceptors and gradually acquired foundational skills and knowledge. The preceptors reported that, over time, they increasingly encouraged the students to work independently, either alone or in pairs. They provided them with opportunities to perform clinical tasks, including basic assessments, wound care, medication administration, disease management, and documentation. Although preceptors often accompanied students during home visits, they intentionally remained in the background to facilitate students’ autonomy and learning.

In the beginning, I mostly observed and tried to learn by watching, but after a couple of weeks, we were given more responsibility and could work more independently in pairs. By the end of the placement, I felt much more confident. I had a better understanding of home care, improved my communication, and felt more prepared to handle unexpected situations on my own. (Student 43)

The preceptors also reported that they took the time to explain and demonstrate practical nursing tasks even when students required additional time to complete their assignments. They viewed themselves as educators and role models. Although the pedagogical intention was to allow students to take the lead in clinical situations, the preceptors occasionally stepped in to assist or take over. These interventions were typically provoked by the preceptors’ concerns about the students’ readiness or competence. From a clinical perspective, the preceptors recognized the potential of nursing students as future colleagues in home healthcare. However, they commonly recommended that students first gain experience in hospital settings before transitioning to home healthcare due to the complexity and autonomy required in the latter.

However, some preceptors admitted that their own lack of experience contributed to uncertainty in their preceptor role:

Even though I wanted the students to manage on their own, I sometimes stepped in too early. Looking back, that was not always because they couldn’t do it, but because I felt unsure as a preceptor (Preceptor 6)

Altogether, these accounts suggest a pattern in which preceptors struggled to balance their intention to support student autonomy with their responsibility to ensure safe practice. Nevertheless, only a few preceptors expressed a need for further formal preceptorship training; instead, they highlighted that peer support may play a more significant role in their development as preceptors. They described growing into their role over time, gaining confidence and experience through repeated interactions with students and collaboration with colleagues:

At first, I wasn’t very confident, but over time, working with students again and again, I’ve grown into the role (Preceptor 45)

Both preceptors’ and students’ accounts revealed that by the end of the four-week clinical training, the students had developed a more nuanced understanding of home-based care, particularly the conditions and responsibilities associated with nursing in this setting. The sustained interactions with the patients provided students with valuable opportunities to observe the continuity of care, understand the complexity of patients’ everyday lives, and appreciate the holistic responsibilities characteristic to home healthcare. In addition, the training facilitated growth in students’ communication, critical reasoning, and clinical decision-making as well as increased confidence in delivering nursing care and managing unexpected situations. The students also reported that over the course of the clinical training, they had not only gained insight into the nursing role within the home healthcare but also developed relationships with patients and their next of kin.

Over time, I got to know the patients and their families, which made it easier to understand their everyday lives. Following the same patients helped me see the continuity of care and how complex their situations can be, and it really showed me what a holistic nursing role in home healthcare involves. (Student 5)

Moreover, some students reported notable development in their collaboration with other students and healthcare professionals. Despite the demands of caring for several patients daily, the students generally felt that collaborating with other students enabled them to perform their assignments without time pressure and constituted a critical factor for effective learning.

While some students expressed a desire for a longer placement to further establish their learning and readiness as nurses, most felt that four weeks were sufficient. Nevertheless, both the students and preceptors acknowledged that the learning process was influenced not only by the duration of the clinical training but also by a range of interacting factors. These factors include the students’ and the preceptors’ individual characteristics, the level of support provided by the university and the preceptors’ supervisor, the overall learning environment, the preceptors’ experience, and the students’ competence and collaboration with peers.

## Discussion

This study shows that clinical practice in a home healthcare setting is a promising and feasible placement option for final‑semester nursing students, where various collaborative strategies can be applied to address both structural and pedagogical challenges. Despite the unique characteristics of home healthcare, the present findings correspond well with earlier studies across different healthcare settings [18, 25, 42, 43, 44]. However, compared with preceptorship in hospital and primary care settings, preceptorship in the home setting presents distinct educational uncertainties and challenges. In this setting, preceptors must balance their professional responsibilities with the unique dynamics of being a guest in the patient’s home [7]. Therefore, they need to adjust their collaborative teaching strategies to the specific patient condition, the ethical dimensions of the situation, and each student’s level of competence. To do so, preceptors can organize preceptorships through collaborative team constellations beyond the traditional two-to-one peer-learning model, adapting the degree and nature of collaboration between preceptors and students to the specific clinical circumstances. The findings indicate that flexible collaborative team-based learning arrangements foster an interactive and reflective learning environment that promotes clinical reasoning, mutual support, and problem-solving in authentic clinical situations. Such collaborative strategies can strengthen students’ and preceptors’ communication skills and facilitate knowledge exchange. In turn, these processes enhance their clinical reasoning and decision-making in interactions with patients and other healthcare professionals [27, 45]. The students expressed experiencing a collegial and inclusive atmosphere within team-based learning arrangements, which previous research has shown to be fundamental to students’ clinical development [46, 47]. Team-based collaborative learning further strengthens trust and enhance students’ confidence in their learning processes. Clear communication, positive interactions, and shared expectations are critical for maintaining team stability. Without these aspects, learning risks becoming fragmented, roles unclear, and patient safety compromised. Phillips et al. [48] highlight that listening to students, providing targeted support and constructive feedback, and offering well-structured assignments (e.g. structured learning activities) are key components of a supportive clinical environment. In this study, the preceptors who considered themselves experienced described themselves as educators and role models. In contrast, those who regarded themselves as novice preceptors tended to adopt a more protective stance and to perceive themselves as learners who needed guidance and support to effectively facilitate student development. These tendencies may stem from an increased sense of responsibility and uncertainty regarding both the clinical and pedagogical dimensions of precepting. While understandable, such tendencies might unconsciously limit students’ opportunities for independent practice, decision‑making, and critical thinking. Accordingly, they underscore the importance of social interaction and scaffolding in learning processes [21]. As preceptorship is a shared responsibility, all clinical staff and managers, not only designated preceptors, must contribute to supporting nursing students during clinical training to maintain an encouraging environment [26].

Although team‑based collaborative preceptorship in home healthcare offers valuable opportunities for developing students’ abilities, it also requires preceptors to constantly balance students’ learning and independence with patient safety and the complexities of providing care in patients’ homes. These considerations are particularly relevant as patients’ homes are designed for living rather than caregiving [49]. Nevertheless, emphasizing the risks does not necessarily imply that care in patients’ homes is unsuitable for clinical training; rather, these risks should be weighed against the benefits of promoting student independence. Doing so requires preceptors to reflect on the structural, pedagogical, and relational conditions required for home healthcare to function as a robust sociocultural learning environment. For instance, conducting independent home visits without the preceptor might provide students with opportunities to develop across multiple aspects of patient care. The findings show that it enhances students’ self-confidence and their application of clinical knowledge by strengthening their problem-solving abilities, critical thinking, and capacity for reflection. Both preceptors and students indicated that students learn through interactions with patients without a preceptor present when accompanied by a peer. They explained that the home as a healthcare setting enables student pairs to gradually develop independence and provides them with opportunities to discuss care-related issues and reflect on the ethical dimensions of care. However, patient safety must be maintained and prioritized, and the preceptors in this study maintained that patients should be asked in advance to approve of students visiting them independently. Establishing a trusting nurse and patient relationship is essential in all healthcare contexts [50], but it may be particularly important when caring for patients in their homes. Therefore, only patients who have explicitly given their consent should be selected for student visits conducted without a preceptor. In this study, such patients were mainly those who were well-known to the preceptors and who were familiar with the students through prior supervised visits. Other patients selected for independent home visits were new patients who merely required a simple procedure that the preceptors were confident their students could handle.

Another important strategy for maintaining patient safety might be to incorporate structured reflection discussions between students and preceptors before and after patient encounters. The findings revealed that pre-planned and well-designed structured learning activities help prepare students ahead of these visits. Such activities support learning by helping students link theory to practice and by fostering the development of clinical skills, confidence, collaboration, and critical thinking [23]. Thus, this collaborative strategy facilitates the gradual development of independence, allowing students to bridge theory and practice over time in increasingly autonomous and meaningful ways.

Despite their potential, collaborative precepting strategies are not without challenges. Preceptors cannot take for granted that students possess fully formed collaborative competencies at the outset of their clinical training [51]. The ability to collaborate effectively within teams is not innate but develops progressively over time [13]. Teamwork and collaboration are general core competencies within nursing practice [52], and these competencies are particularly relevant for home healthcare, where interdisciplinary collaboration is not only prevalent but essential for ensuring safe, efficient, and high‑quality patient care [53]. Therefore, nursing students ought to be provided with structured opportunities to develop and apply collaborative skills, first through campus‑based learning activities [12] and later during their clinical training [25]. Strengthening such competencies not only supports student collaborative learning but also prepares them for meeting the demands of person‑centred community- and team-based care.

## Conclusion

To meet the demands of a shifting healthcare landscape, graduated nurses must be prepared to deliver high-quality, safe, and person-centred care also in the patients’ homes. Overall, the findings if this study indicate that home healthcare is a meaningful and feasible setting for clinical training for final-semester nursing students. The home setting provides students with the opportunity to care for patients with complex healthcare needs, particularly older, frail, and multimorbid individuals. Home-based training complements hospital-based training by fostering students’ autonomy, clinical decision-making, and interprofessional collaboration. Diverse collaborative pedagogical strategies can support both precepting and student learning, but they require preceptors to balance fostering student autonomy with ensuring patient safety. Therefore, students’ independent home visits should be limited to situations that do not involve complex treatments or assessments of critically ill patients.

Furthermore, the findings suggest that structured learning activities and collaboration between preceptors and students offer mutual scaffolding within their respective zones of proximal development. Several scaffolding strategies were recognized in our findings. First, the redistribution of precepting responsibilities across several preceptors is a collective scaffolding structure that reduces the workload placed on individual preceptors while simultaneously broadening students’ learning opportunities through exposure to varied clinical perspectives and professional expertise. Second, pairing novice preceptors with experienced colleagues is another form of scaffolding that promotes mutual learning and strengthens the pedagogical capacity of the precepting team. Experienced preceptors offer stability in both the team and the clinical learning environment, implement collaborative precepting strategies, and support professional behaviour. Consequently, close co-operation between healthcare organizations and universities is essential to enhance preceptors’ competence and confidence in the precepting role. Therefore, further research should explore the long-term effects of team-based preceptorship on student competence, preceptors’ professional experiences, patient safety, and care quality. It should also incorporate the perspectives of patients and their next of kin.

## Supplementary Information

Below is the link to the electronic supplementary material.

Supplementary Material 1

Supplementary Material 2

Supplementary Material 3

## Data Availability

No qualitative data analysis software or artificial intelligence (AI) tools were used in the generation, analysis, or interpretation of the data. The datasets analysed in the current study are available from the corresponding author on reasonable request. The author confirms that all data generated or analysed during this study are included in this published article.

## References

[1] World Health Organization. Global strategy and action plan on ageing and health. Geneva: World Health Organization; 2017.

[2] Sogstad M, Hellesø R, Skinner MS. The development of a new care service landscape in Norway. Health Serv Insights. 2020;13:1178632920922221. 10.1177/1178632920922221. PMC7285946 32565676

[3] Van Eenoo L, van der Roest H, Onder G, Finne-Soveri H, Garms-Homolova V, Jonsson PV, et al. Organizational home care models across Europe: a cross-sectional study. Int J Nurs Stud. 2018;77:39–45. 10.1016/j.ijnurstu.2017.09.013. 29032349

[4] Eckerblad J, Theander K, Ekdahl A, Unosson M, Wirehn AB, Milberg A, et al. Symptom burden in community-dwelling older people with multimorbidity: a cross-sectional study. BMC Geriatr. 2015;15:1. 10.1186/1471-2318-15-1. PMC4292813 25559550

[5] Larsson Gerdin A, Hellzén O, Kjällman Alm A, Holmström Rising M. Encountering patients in their homes: a qualitative study of home care nurses’ experiences. Nord J Nurs Res. 2023;43(1). 10.1177/20571585231158950.

[6] Turjamaa R, Hartikainen S, Kangasniemi M, Pietilä AM. Living longer at home: a qualitative study of older clients’ and practical nurses’ perceptions of home care. J Clin Nurs. 2014;23(21–22):3206–17. 10.1111/jocn.12569. 25453125

[7] Heggestad AKT, Magelssen M, Pedersen R, Gjerberg E. Ethical challenges in home-based care: a systematic literature review. Nurs Ethics. 2021;28(5):628–44. 10.1177/0969733020968859. 33334250

[8] Andersson H, Lindholm M, Pettersson M, Jonasson L-L. Nurses’ competencies in home healthcare: an interview study. BMC Nurs. 2017;16:65. 10.1186/s12912-017-0264-9. PMC5693583 29176934

[9] Melby L, Obstfelder A, Hellesø R. We tie up the loose ends: homecare nursing in a changing health care landscape. Glob Qual Nurs Res. 2018;5:2333393618816780. 10.1177/2333393618816780. PMC6295756 30574532

[10] Brenne BA, Hedlund M, Ingstad K. Exploring home-based care nurses’ mindset for nursing practices: a phenomenological study. BMC Nurs. 2022;21:291. 10.1186/s12912-022-01068-w. PMC9623960 36316738

[11] Andrade AM, Silva KL, Seixas CT, Braga PP. Nursing practice in home care: an integrative literature review. Rev Bras Enferm. 2017;70(1):199–208. 10.1590/0034-7167-2016-0214. 28226061

[12] Stenberg M, Bengtsson M, Mangrio E, Carlson E. Collaboration between first year undergraduate nursing students: a focused ethnographic study. Nurse Educ Pract. 2022. 10.1016/j.nepr.2022.103427. 35994802

[13] Stenberg M, Bengtsson M, Mangrio E, Carlson E. Supporting each other towards independence: a narrative analysis of first-year nursing students’ collaborative process. Nurs Inq. 2024;31:e12627. 10.1111/nin.12627. 38351462

[14] Palander S, Haapa J, Juntunen T, Lim S, Zhou W, Tomietto M, Mikkonen K. The effectiveness of peer learning interventions in nursing students’ clinical practice: a systematic review. Nurse Educ Pract. 2026;92:104781. 10.1016/j.nepr.2026.104781. 41785638

[15] Manninen K. Experiencing authenticity – the core of student learning in clinical practice. Perspect Med Educ. 2016;5:308–11. 10.1007/s40037-016-0294-0. PMC5035280 27638390

[16] Hobenu KA, Adefuye AO, Naab F, Nyoni CN. Strategies to enhance clinical teaching and learning in undergraduate nursing education: a scoping review. PLoS ONE. 2025;20(6):e0305789. 10.1371/journal.pone.0305789. PMC12151355 40493639

[17] Mager DR. Home care: promoting knowledge and creating impact among nursing students. J Community Health Nurs. 2021;38(2):120–9. 10.1080/07370016.2021.1887567. 33949263

[18] Markowski M, Bower H, Essex R, Yearley C. Peer learning and collaborative placement models in health care: a systematic review and qualitative synthesis of the literature. J Clin Nurs. 2021;30(11–12):1519–41. 10.1111/jocn.15661. 33461240

[19] Topping KJ. Trends in peer learning. Educ Psychol. 2005;25(6):631–45. 10.1080/01443410500345172.

[20] Stenberg M, Carlson E. Swedish student nurses’ perception of peer learning as an educational model during clinical practice in a hospital setting: an evaluation study. BMC Nurs. 2015;14:48. 10.1186/s12912-015-0098-2. PMC4592542 26435698

[21] Vygotsky LS. In: Cole M, Jolm-Steiner V, Scribner S, Souberman E, editors. Mind in society: the development of higher psychological processes. Cambridge, MA: Harvard University Press; 1978. 10.2307/j.ctvjf9vz4.

[22] Nygren F, Carlson E. Preceptors’ conceptions of a peer learning model: a phenomenographic study. Nurse Educ Today. 2017;49:12–6. 10.1016/j.nedt.2016.10.015. 27865125

[23] Stenberg M, Bengtsson M, Mangrio E, Carlson E. Preceptors’ experiences of using structured learning activities as part of the peer learning model: a qualitative study. Nurse Educ Pract. 2020;42:102668. 10.1016/j.nepr.2019.102668. 31778874

[24] Hublin L, Koivisto JM, Lyyra M, Haavisto E. Learning collaborative clinical reasoning in healthcare education: an integrative review. J Prof Nurs. 2023;49:126–34. 10.1016/j.profnurs.2023.09.011. 38042546

[25] Sandvik AH, Karlsson P, Zetterman A, Eskilsson C. Nursing students’ experiences of peer learning in a dedicated educational unit in municipal home healthcare: a phenomenological study. Nord J Nurs Res. 2021;41(4):224–32. 10.1177/2057158520966949.

[26] Jassim T, Carlson E, Bengtsson M. Preceptors’ and nursing students’ experiences of using peer learning in primary healthcare settings: a qualitative study. BMC Nurs. 2022;21(1):1–10. 10.1186/s12912-022-00844-y. PMC8939121 35313874

[27] Nilsson C, Lindberg B. Demanding yet supportive and developing: nurse preceptors’ experiences of peer learning in nursing education in municipal elderly care homes. Nord J Nurs Res. 2024;44. 10.1177/20571585241231297.

[28] Oermann MH, Shellenbarger T, Gaberson KB. Clinical teaching strategies in nursing. 6th ed. New York: Springer Publishing Company; 2022. 10.1891/9780826167057.

[29] Shochet RB, Colbert-Getz JM, Levine RB, Wright SM. Gauging events that influence students’ perceptions of the medical school learning environment: findings from one institution. Acad Med. 2013;88(2):246–52. 10.1097/ACM.0b013e31827bfa14. 23269291

[30] Wilson AD, Onwuegbuzie AJ, Manning LP. Using paired depth interviews to collect qualitative data. Qual Rep. 2016;21(9):1549–73. 10.46743/2160-3715/2016.2166.

[31] Acocella I. The focus groups in social research: advantages and disadvantages. Qual Quant. 2012;46:1125–36. 10.1007/s11135-011-9600-4.

[32] Boud D. Introduction: making the move to peer learning. In: Boud D, Cohen R, Sampson J, editors. Peer learning in higher education: learning from and with each other. London: Kogan Page; 2001. pp. 1–17. 10.4324/9781315042565.

[33] Kolb DA. Experiential learning: experience as the source of learning and development. Englewood Cliffs, NJ: Prentice Hall; 1984.

[34] Gibbs G. Learning by doing: a guide to teaching and learning methods. Oxford: Oxford Polytechnic; 1988.

[35] Lipnevich AA, Mattern K, Feddock C. Formative assessment and feedback in medical education: a practical guide. AMEE Guide 189 Med Teach. 2025. 10.1080/0142159X.2025.2569623. 41134874

[36] Malterud K, Siersma VD, Guassora AD. Sample size in qualitative interview studies: Guided by information power. Qual Health Res. 2016;26(13):1753–60. 10.1177/1049732315617444. 26613970

[37] Roberts S-J, Saylor MA, Ivynian S, DiGiacomo M. Data saturation and information power: sample size in qualitative research-when is enough and how to assess? Eur J Cardiovasc Nurs. 2026;00:1–6. 10.1093/eurjcn/zvag046. 41693424

[38] DiCicco-Bloom B, Crabtree BF. The qualitative research interview. Med Educ. 2006;40(4):314–21. 10.1111/j.1365-2929.2006.02418.x. 16573666

[39] Braun V, Clarke V. Using thematic analysis in psychology. Qual Res Psychol. 2006;3(2):77–101. 10.1191/1478088706qp063oa.

[40] Guba EG, Lincoln YS. Fourth generation evaluation. Newbury Park, CA: SAGE; 1989.

[41] World Medical Association. World Medical Association Declaration of Helsinki: ethical principles for medical research involving human subjects. J Korean Med Assoc. 2014;57(11):899–902. 10.5124/jkma.2014.57.11.899. 19886379

[42] Vuckovic V, Karlsson K, Sunnqvist C. Preceptors’ and nursing students’ experiences of peer learning in a psychiatric context: a qualitative study. Nurse Educ Pract. 2019;41:102627. 10.1016/j.nepr.2019.102627. 31751904

[43] Kjällquist-Petrisi A, Hommel A. Preceptors’ experiences of peer learning in an intensive care unit. Nurse Educ Pract. 2021;54:103086. 10.1016/j.nepr.2021.103086. 34091102

[44] Hammar S, Carlson E, Persson K. Nurse anesthetist students’ experiences of peer learning in clinical education: a qualitative study. J Prof Nurs. 2023;44:62–8. 10.1016/j.profnurs.2022.12.002. 36746601

[45] Radkowitsch A, Fischer MR, Schidmaier R, Fischer F. Learning to diagnose collaboratively: validating a simulation for medical students. GMS J Med Educ. 2020;37(5):Doc51. 10.3205/zma00. PMC7499460 32984510

[46] Rodríguez-García MC, Gutiérrez-Puertas L, Granados-Gámez G, Aguilera-Manrique G, Márquez-Hernández VV. The connection of the clinical learning environment and supervision of nursing students with student satisfaction and future intention to work in clinical placement hospitals. J Clin Nurs. 2021;30(7–8):986–94. 10.1111/jocn.15642. 33432645

[47] Hilli Y, Sandvik AH. A Nordic model for learning compassionate care in clinical education. Nurse Educ Today. 2020;90:104454. 10.1016/j.nedt.2020.104454. 32371297

[48] Phillips KF, Mathew L, Aktan N, Catano B. Clinical education and student satisfaction: an integrative literature review. Int J Nurs Sci. 2017;4(2):205–13. 10.1016/j.ijnss.2017.03.004. PMC6626144 31406743

[49] Shahrestanaki SK, Rafii F, Najafi Ghezeljeh T, Farahani MA, Majdabadi Kohne ZA. Patient safety in home health care: a grounded theory study. BMC Health Serv Res. 2023;23:467. 10.1186/s12913-023-09458-9. PMC10171141 37165357

[50] Henriksen SL, Eekholm S, Poulsen I, Kristensson J. The lived experience of the trusting nurse–patient relationship among patients admitted to internal medicine wards. J Adv Nurs. 2025 Nov;3. 10.1111/jan.70344. PMC13267437 41178808

[51] Ha L, Janssen J, Wubbels T. Collaborative learning practices: teacher and student perceived obstacles to effective student collaboration. Camb J Educ. 2018;48(1):103–22. 10.1080/0305764X.2016.1259389.

[52] Cronenwett L, Sherwood G, Barnsteiner J, Disch J, Johnson J, Mitchell P, et al. Quality and safety education for nurses. Nurs Outlook. 2007;55(3):122–31. 10.1016/j.outlook.2007.02.006. 17524799

[53] Goosen S. The importance of teamwork in nursing. Prof Nurs Today. 2015;19:4–6.

