# Supplementary material for: Preceptors’ and nursing students’ experiences of collaborative pedagogical strategies in home healthcare: a qualitative study
Source: BMC Nurs. 2026 Nov 11;25:1106. doi: 10.1186/s12912-026-05651-3 (PMC13757084; doi:10.1186/s12912-026-05651-3)
Supplement: Supplementary file 1 [file 12912_2026_5651_MOESM1_ESM.pdf]

Supplementary file 1

TASKS FOR NURSING STUDENTS IN HOME HEALTH CARE

Structured learning activities and topics for reflection - optional to undertake individually or with a peer.

STUDY WEEK	THEME	REFLECTION FOR UNDERSTANDING	STRUCTURED LEARNING ACTIVITIES
**Week 1**	Introduction of the organization	To enhance understanding of involved healthcare providers' responsibilities.<br><br>• Routines when the patient has multiple care providers<br>• What does it mean for the patient to have multiple care providers?	1. ***Nursing process - MANDATORY!***<br><br>2. Receive a patient discharged from hospital<br>3. Coordinated Individual Care Plan<br>4. Palliative care Part 1<br>5. Safe care at home Part 1
**Week 2**	Digital tools	To develop understanding of communication between different health care professionals as well as patients using digital tools.	6. Senior Alert<br>7. Urinary incontinence
**Week 3**	Person-centred care	To plan and provide person-centred care in patients' homes.	8. Wound care Part 1<br>9. Wound care Part 2<br>10. Palliative care Part 2<br>11. Medication management Part 1
**Week 4**	Leadership and collaboration	To develop leadership and collaborative skills with a focus on nursing care.<br><br>• Nurses work environment<br>• Patients' technical care equipment<br>• Patients different living condition	12. Domestic violence<br>13. Palliative care Part 3<br>14. Medication management Part 2<br>15. Safe care at home Part 2
