# Supplementary material for: Preceptors’ and nursing students’ experiences of collaborative pedagogical strategies in home healthcare: a qualitative study
Source: BMC Nurs. 2026 Nov 11;25:1106. doi: 10.1186/s12912-026-05651-3 (PMC13757084; doi:10.1186/s12912-026-05651-3)
Supplement: Supplementary file 2 [file 12912_2026_5651_MOESM2_ESM.pdf]

Supplement file 2

The Nursing Process – a mandatory Structured Learning Activity

Activity related to the Learning Outcomes in Syllabus and assessment based on the AssCe form (Assessment of Clinical Education*, item 1-5, 6-10, 16-18<br><br>* Assessment of Clinical Nursing Education with the AssCE Tool - Högskolan i Gävle	Level<br><br>Year 3

Planning

- Read relevant literature and chapters in the Swedish National Healthcare Handbook, as well as the article; Höglander J, Eklund JH, Spreeuwenberg P, Eide H, Sundler AJ, Roter D, Holmström (2020) Exploring patient-centered aspects of home care communication: a cross-sectional study, IK.BMC Nurs. 2020 Sep 29;19:91. doi: 10.1186/s12912-020-00483-1.
- Identify a patient who has complex nursing needs

Execution

Begin with a conversation with the patient and use relevant assessment instruments when appropriate.

- Identify the patient's physical, psychological, social, and existential needs and resources.
- Explore the patient's expectations regarding nursing care. What does the patient want help with, and what is important to them?
- Prioritize one nursing need that is particularly important to the patient and report this to your preceptor.

Establish a care plan, preferably together with the patient.

- The care plan should include a nursing diagnosis, clearly defined nursing goals, and evidence-based nursing interventions, while incorporating the patient's resources and expectations.
- Are there family members present in the home? To what extent do they wish to be involved?
- Report back to your preceptor and then document the care plan. How and when should the nursing interventions be evaluated?

Implement and evaluate the established care plan.

- Carry out relevant nursing interventions and involve the patient, family members and other healthcare professionals as needed.
- Evaluate the outcomes of the nursing care and document.

Reflection

Reflect together with a peer and/or preceptor on your experience of the patient interaction, any challenges encountered, areas for further learning, and how the home setting influenced the nursing care.

Instructions for the Preceptor

Introduce the activity during the first week and support the student in working with the nursing process throughout the entire clinical placement period. Assist the student in selecting a patient with complex nursing needs whom they can follow up over time. Engage in reflective discussions with the student using Kolb's cycle of reflective practice. Discuss the nursing process based on what the student observed as well as what may have been overlooked. Contribute additional perspectives and insights that you consider important for the student's learning.
