# Supplementary material for: Preceptors’ and nursing students’ experiences of collaborative pedagogical strategies in home healthcare: a qualitative study
Source: BMC Nurs. 2026 Nov 11;25:1106. doi: 10.1186/s12912-026-05651-3 (PMC13757084; doi:10.1186/s12912-026-05651-3)
Supplement: Supplementary file 3 [file 12912_2026_5651_MOESM3_ESM.pdf]

Semi-Structured Interview Guide

Precepting process:
Can you tell me about your experience of the precepting process during the placement?

Collaboration:
Can you describe your collaboration with other students and/or preceptors during the placement?

Reflection:
Can you tell me about any opportunities you had to reflect during the placement?

Learning environment:
How would you describe the learning environment you were part of?

Structured learning activities:
Can you describe your experiences from the learning activities you participated in?

Overall placement experience:
Can you tell me about your overall experience of the placement?

Follow-up prompts:
Can you elaborate on that? Could you clarify what you mean? Can you give an example? What worked well or less well? How did it affect your learning/precepting?
